# Examining the supply and need of health workforce in Ethiopia: A foundation for strategic investment in human resources for health

**DOI:** 10.1016/j.puhip.2026.100784

**Published:** 2026-03-28

**Authors:** Jemal Mohammed Ali, Firew Ayalew, Zeine Abosse, Tegbar Yigzaw Sendekie, Shelemo Shawula, Fikadu Asrat, Mezgebu Yitayal, Habtamu Demissie, Biniyam Biresaw, Jelle Stekelenburg

**Affiliations:** a University of Groningen (URG) University Medical Centre Groningen (UMCG), the Netherlands; b Freelance Reseracher Ethiopia, Ethiopia; c URG/UMCG the Netherlands; d Mastercard Foundation, Canada; e MOH Ethiopia, Ethiopia; f Bahir Dar University, Ethiopia; g University Medical Centre Groningen/University of Groningen, the Netherlands

**Keywords:** Health workforce, Labour market analysis, Fiscal space, Human resources for health, Ethiopia, Universal health coverage

## Abstract

**Objectives:**

We conducted a Health Labour Market Analysis (HLMA) to evaluate the alignment of health workforce supply with population health needs and fiscal sustainability through 2030.

**Study design:**

A quantitative study design using secondary data was based on the World Health Organization (WHO) HLMA Framework and the 2021 WHO HLMA Guidebook.

**Methods:**

Quantitative data were gathered from the Human Resources Information System (HRIS), professional associations, training institutions, and national accounts, supplemented by grey literature and stakeholder consultations. Workforce supply and demand projections for 2024–2030 considered an attrition rate of 3.5%, a 20% unemployment rate for new graduates, and an 80% absorption rate. Financial analyses were aligned with Gross Dometic Product (GDP) and fiscal projections from the World Bank, International Monetary Fund (IMF), and the National Bank of Ethiopia. Data quality assurance included multi-level validation using a standardized Ministry of Health (MOH) tool, with outlier checks and stakeholder verification.

**Results:**

The supply of health professionals is projected to increase steadily, reaching approximately 74,693 nurses, 30,980 midwives, and 25,576 general practitioners by 2030. Despite these gains, significant shortages persist relative to Essential Health Services Package (EHSP)–based requirements, particularly among medical specialists, nurses, anesthetists, and laboratory professionals. Financial analysis indicates that cumulative fiscal space is projected at USD 945 million by 2030, while the cost of employing the available workforce is estimated at USD 1.08 billion, and the EHSP-aligned requirement at USD 1.8 billion. This results in an annual financing gap of USD 20–30 million for workforce absorption and over USD 800 million relative to service needs.

**Conclusions:**

Ethiopia's HLMA highlights gaps between workforce supply, health needs, and fiscal capacity. Despite an increase in graduates, unemployment persists. Improving Human Resource for Health (HRH) governance, expanding fiscal resources, and ensuring fair deployment are vital for effective workforce utilization and advancing universal health coverage.

## 1 Introduction

The global health workforce crisis remains one of the most pressing challenges to achieving universal health coverage (UHC) and Sustainable Development Goal (SDG) 3 on “Good Health and Well-being.” The World Health Organization (WHO) projects a global shortage of 11 million health workers by 2030, with the greatest deficits concentrated in low- and middle-income countries (LMICs) [[1], [2], [3]]. Addressing this shortfall will require substantial investments, estimated at over half of the global financing needed to meet SDG 3 targets [2]. The COVID-19 pandemic had further exposed the systemic weaknesses, placing extraordinary pressure on health workers and contributing to attrition through burnout, infection, and migration [4,5].

In the African region, the shortage is particularly acute, with an estimated 6.1 million health workers lacking by 2030 [3]. This gap threatens progress in maternal and child health, prevention and control of infectious disease, and management of non-communicable disease. Although many African countries have expanded training capacity for doctors, nurses, and midwives, absorption into the public sector has lagged due to fiscal constraints. This has created a paradox of trained but unemployed health professionals [6,7]. Such mismatches between workforce supply and labour market demand highlight the urgent need for more robust health labour market analyses (HLMA) to inform evidence-based workforce policies.

Within this regional context, Ethiopia faces significant health workforce gaps that affect service delivery and health outcomes. Despite considerable expansion of pre-service education over the past two decades, shortages, inequitable distribution, and performance challenges persist. In 2022/23, Ethiopia allocated only 8.1% of its national budget to health—well below the 15% target set under the Abuja Declaration [8]. Although the overall number of health professionals has increased [9], shortages remain across critical cadres [10], with inequitable regional distribution [11], high attrition rates [12], and suboptimal productivity [13].

The consequences of these workforce constraints are particularly evident in reproductive, maternal, and newborn health, Shortages of skilled midwives and emergency obstetric care providers contribute to preventable maternal deaths. Ethiopia's high maternal mortality ratio is closely linked to limited access to skilled birth attendants and inadequate emergency obstetric and newborn care services [[14], [15], [16]]. These patterns illustrate how insufficient workforce availability and poor distribution undermine safe motherhood efforts and broader health system performance.

The Ministry of Health (MOH), in collaboration with development partners, has initiated reforms to improve the availability, quality, and equitable deployment of health workers [17]. However, persistent labour market mismatches and fiscal limitations continue to hinder effective workforce absorption and service expansion.

Ethiopia-specific evidence remains limited in broader regional analyses. For instance, the WHO African Region (AFRO) Decade Review of the Health Workforce (2013–2022) provides important regional insights but offers only limited analysis specific to Ethiopia [18]. To generate the granular data required for national planning, Ethiopia requires a comprehensive Health Labour Market Analysis (HLMA) guided by the updated WHO HLMA framework [19], which applies an economic lens to assess workforce supply, demand, and financing capacity.

This study uses validated national data to project workforce supply, demand, and fiscal space through 2030; examine shortages alongside graduate unemployment; and assess policy options. It also incorporates a needs-based analysis aligned with Ethiopia's Essential Health Services Package (EHSP) [20]. By triangulating supply, demand, and need, the HLMA identifies labour market mismatches and financing constraints, providing an evidence base for strategic workforce planning and sustainable investment.

### 1.1 Objectives

This study aims to.

1. Project trends in Ethiopia's health workforce supply from 2025 to 2030.
2. Estimate need-based health workforce requirements aligned with the Essential Health Services Package.
3. Assess gaps between projected workforce supply and population health needs.
4. Estimate the financial resources required to address these gaps between 2025 and 2030.

## 2 Methods

### 2.1 Study design and framework

**Fig. 1:**
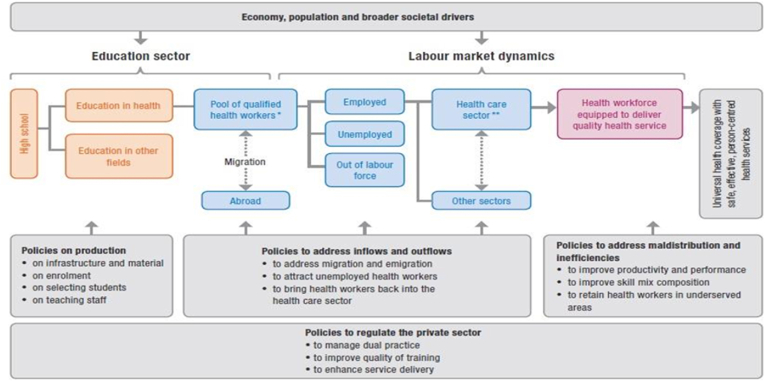
Comprehensive Health Labour Market Framework for UHC Adapted from: Sousa et al., 2013 [24].

A quantitative study design was employed to conduct a comprehensive national-level analysis using the World Health Organization (WHO) HLMA framework [19] (Fig. 1), integrated with Ethiopia's Essential Health Services Package (EHSP, 2019) [20]. This approach enables systematic examination of health labour market dynamics and supports evidence-based assessment of workforce imbalances. The WHO HLMA framework is an economic and systems-based approach that examines the interaction between health workforce supply, labour market demand, population health needs, and fiscal capacity. It helps identify labour market mismatches—such as the coexistence of workforce shortages and graduate unemployment—and supports evidence-based workforce and financing policies [19,21]. We used the WHO HLMA framework operationalized through an Excel-based simulation model because it applies established WHO methodologies for workforce supply projections, needs-based estimates, and fiscal space analysis. This approach has been widely used in WHO-supported HLMA and HRH planning studies in LMICs, ensuring methodological consistency and policy relevance [13,22,23].

Using this framework, the study systematically analyzed health workforce production, stock, availability, skill mix, and attrition, as well as labour market inflows and outflows (Fig. 1). Relevant national policies, strategies, and guidelines were reviewed to contextualize findings and inform interpretation.

Stakeholder meetings and workshops with MOH directors, policymakers, and program implementers were held to gather insights and assess data availability.

### 2.2 Study setting and population

The study covered Ethiopia, a sub-Saharan African country with an estimated population of 132 million in 2024, projected to reach 150 million by 2030 [25]. Ethiopia's health system is organized into three tiers—primary, secondary, and tertiary care. There is a strong emphasis on primary health care, which is delivered through health centers and health posts. Health Extension Workers (HEWs) and mid-level healthcare professionals, such as health officers, nurses, and midwives, play a central role in providing essential clinical services. The mid-level health professionals are those who bridge the gap between community-based care and physician-level care, particularly in rural and underserved areas.

### 2.3 Cadres included

The analysis included doctors, nurses, midwives, health officers, anaesthesia professionals, medical laboratory personnel, pharmacists, HEWs, and administrative/support staff, as defined in the EHSP [13]. These cadres collectively underpin the delivery of essential health services, including diagnosis, treatment, emergency care, maternal and child health, pharmaceuticals, diagnostics, community-based prevention, and health system management [1,3,[22], [23], [26], [27], [28], [29], [30]]. Including both clinical and non-clinical cadres ensured a system-wide perspective on workforce requirements.

### 2.4 Data sources and assumptions

Data were collected using a standardized HLMA tool aligned with the WHO HLMA Guidebook and endorsed by MOH. Sources included the Human Resource Information System (HRIS), professional associations, training institutions, national administrative reports, and international databases (Table 1). Baseline workforce stock for 2024 was validated through consultations with MOH directorates and regional health bureaus.

**Table 1:** Data sources for the analysis.

Dimension for the Key Variable	Parameter	Data Source
Workforce stock	Number of health professionals by cadre, sex, and location	HRHIS (MOH)
Workforce performance and trends	Annual workforce updates and sector performance	Health Sector Annual Performance Reports; Workforce Bulletins
Workforce production	Enrollment and graduation rates	Ministry of Education (MoE); Higher Education Institutions
Service delivery and facility readiness	Facility-level staffing, service delivery indicators	HMIS/DHIS2; SARA Reports
Professional regulation	Licensing, registration, and membership data	Professional Associations
Population demographics	Population projections for density and service needs	UNFPA; Central Statistical Agency (CSA)
Financial and macroeconomic context	Health expenditure, wage bill, GDP, fiscal space	National Health Accounts (2019/20); Government Budget Portfolio; World Bank; IMF; Ministry of Finance
Supplementary evidence	Contextual and analytical information	Grey literature: Reports from WHO, USAID, World Bank, and other partners

Key assumptions included a 5–10% pre-service training dropout rate, an average graduate absorption rate of 80% [31], and an annual attrition rate of 3.5% based on national reports and WHO benchmarks [32,33]. Population projections were derived from UNFPA estimates [25]. Need-based requirements followed WHO density thresholds for doctors, nurses, and midwives and EHSP staffing norms for other cadres.

Macroeconomic assumptions for fiscal space analysis were aligned with Ministry of Finance projections, National Health Accounts data, and National Bank of Ethiopia exchange rate assumptions, incorporating inflation-adjusted wage estimates.

### 2.5 Analytical models

We applied an empirical framework to integrate health workforce supply, needs, and financial feasibility [19,21,34]. Workforce supply was estimated using a stock-and-flow approach, needs were derived from EHSP service standards and WHO WISN methods [22,23,35], and financial feasibility was assessed by comparing projected workforce costs with available public and private sector fiscal space [19,36,37]. Mean projections were generated for the period 2024–2030, accompanied by 95% confidence intervals to reflect uncertainty related to attrition, graduate absorption, economic growth, and productivity assumptions.

**Fig. 2:**
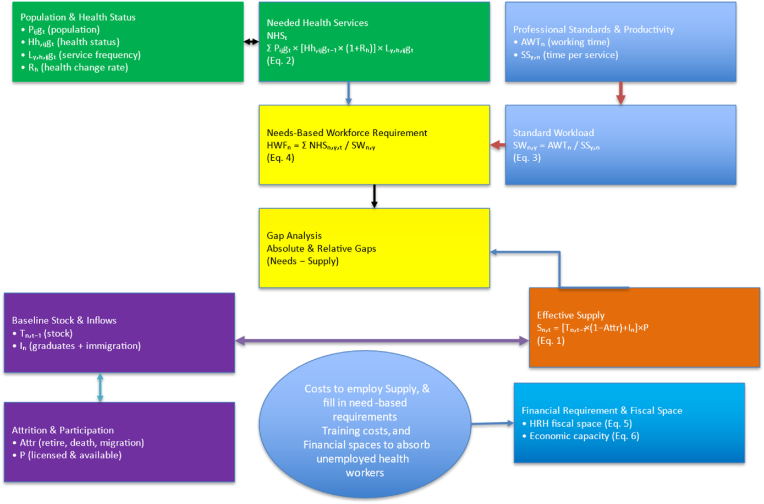
How HLMA Equations Were Applied in This Study (Ethiopia) Source: adapted from Asamani et al. [22,23].

The HLMA was implemented using an equation-based workflow in which separate models estimated workforce supply, needs-based requirements, and fiscal space. Each component was operationalized through standardized equations reflecting stock-and-flow supply dynamics, service-based workforce needs, and projected financing capacity. Fig. 2 illustrates how the study operationalized this framework by sequentially applying supply-based (Equation (1)), need-based (Equations (2)–(4)), and financial space models (Equations (5) and (6)) to generate workforce gaps and financing requirements for Ethiopia

#### 2.5.1 Supply-based model

The supply-based model we used aligns with methods from previous HLMA exercises. Similar models that incorporate baseline stock, inflows, and attrition have been applied in WHO HLMA and workforce studies [22,23].

*Sn,t* = *[Tn,t -1 × (1 – Attr) + In ] × P***→** equation (1)

Where.

Sn,t = the supply of health workers of category n, at time t,

Tn,t = Aggregate stock of health workers of category n at time t,

1-Attr = represents the attrition of health workers – *those who have retired, passed away, faced health challenges that prevented them from working, or migrated out*.

*In* = represent the inflow of health workers of category n trained domestically or may be immigrating from another country.

P = proportion of health workers willing to participate in professional practices.

#### 2.5.2 Need-based model

**Equation 2**

NHS_t_ = ∑ Pᵢⱼg_t_ × [Hh,ᵢⱼg_t-1_ × (1 + R_h_)] × Lᵧ,_h_,ᵢⱼg_t_ - Equation 2

• NHS_t_: needed health services for a population at time t
• Pᵢⱼg_t_: population by age, gender, and location
• Hh,ᵢⱼg_t_: proportion of population with health status h
• Lᵧ,_h_,ᵢⱼg_t_: frequency of required service type y
• R_h_: rate of change in health status

**Equation (3):**

SWn,y = AWTn / SSy,n

• SWn,y: standard workload for cadre n, service y
• AWTn: annual available working time of cadre n
• SSy,n: service standard (time per activity)

**Equation (4):**

Needs-based HWF_n_,ᵧ = ∑ NHS_n_,ᵧ,_t_ / SW_n_,ᵧ

• NHS_n_,ᵧ,_t_: needed services of type y for cadre n at time t
• SW_n_,ᵧ: standard workload

Source: adapted from Asamani et al. [22,23].

#### 2.5.3 Financial requirement model

⁃ **Public Sector Economic Demand (HRH Fiscal Space) for the year, i** = (GGHE as % GDP_i_ ∗ Nominal GDP Values_i_) ∗ HRH Expenditure as % GGHE _i_ Equation … (5)

⁃ **Cumulative Economic Capacity for the year,** i = Public Sector Fiscal Space _i_ ∗ (1+proportion of private sector HRH employment) Equation … (6)

Where:

i = target year.

GGHE = General Government Health Expenditure.

GDP = Gross Domestic Product.

### 2.6 Data validation

Data were validated through cross-checks across sources, internal consistency reviews, MOH technical review, and stakeholder consultations.

## 3 Results

### 3.1 Health workforce supply projections

The total health workforce included in this study is projected to increase from 313,097 in 2024 to 404,196 in 2030, representing an overall growth of 29%. Growth rates vary across cadres. General practitioners, pharmacists, medical specialists, and HEWs exhibit the fastest expansion, reflecting continued investments in pre-service training. Nurses, who constitute the largest cadre, grow by approximately 12%, while administrative and support staff expand more slowly, particularly at the diploma level (Table 2).

**Table 2:** Projected Health Workforce Supply, Ethiopia (2025–2030) with 95% confidence Interval.

Professions	2024	2025	2026	2027	2028	2029	2030
Medical Specialist	7104	7745 (7354 –8292)	8372 (7992 –8922)	8986 (8614–9540)	9588 (9220.1–10150)	10176 (9816 –10742)	10753 (10406–11318)
General Practitioners (Non-Specialist)	14222	16216 (15397 –17363)	18168 (17343–19361)	20080 (19249–21317)	21951 (21109–23237)	23783 (22942 –25104)	25576 (24750–26920)
Non-physician Clinicians (Health Officer)	17780	18763 (17816–20090)	19726 (18829–21021)	20667 (19811–21941)	21590 (20761 –22855)	22493 (21698 –23742)	23377 (22621–24605)
Nurses	66917	68282 (64834–73110)	69620 (66455–74191)	70929 (67991–75299)	72210 (69438–76440)	73465 (70868–77546)	74693 (72278–78616)
Midwives	22786	24225 (23002–25938)	25634 (24469–27317)	27013 (25894–28678)	28364 (27275–30025)	29686 (28637–31335)	30980 (29979–32607)
Anaesthesia Professionals	4912	5069 (4813–5427)	5221 (4984–5564)	5372 (5149–5703)	5518 (5306–5842)	5662 (5462–5976)	5801 (5614–6106)
Medical Laboratory	14796	15692 (14899.9–16802.1)	16571 (15817–17659)	17431 (16709.–18505)	18272 (17571–19343)	19097 (18422–20158)	19904 (19261–20949)
Pharmacists	18281	20091 (19076–21512)	21863 (20869–23299)	23598 (22620–25052)	25295 (24325–26777)	26959 (26006–28456)	28586 (27662–30088)
Health Extension Workers	64206	69491 (65981–74405)	74665 (71271.0–79567.0)	79730 (76428–84642)	84689 (81438–89650)	89544 (86378–94518)	94296 (91248–99248)
Administrative and Support Professionals (Degree and Masters)	15250	16057 (15246–17192)	16848 (16082–17954)	17621 (16891–18707)	18379 (17674–19456)	19121 (18445–20183)	19847 (19205–20889)
Administrative and Support Staff (Diploma and Below)	66842	67464 (64056–72234)	68072 (64978–72542)	68668 (65825-72900)	69252 (66594–73309)	69824 (67355–73703)	70383 (68108–74080)
**Total**	**313,097**	**329,096**	**344,761**	**360,095**	**375,109**	**389,809**	**404,196**
**Net Increase**		**5%**	**5%**	**4%**	**4%**	**4%**	**4%**

### 3.2 Need-based workforce requirements

Need-based requirements aligned with the EHSP increase steadily across all cadres, rising by approximately 15–20% over the projection period. Nurses account for the largest absolute need, followed by medical specialists, laboratory professionals, and general practitioners. Midwives, health officers, and anaesthesia professionals also show consistent increases, reflecting rising service demand driven by population growth and UHC targets (Table 3).

**Table 3:** Projected Health Workforce Requirements based on Health Needs Approach (Essential Health Services Package) with 95% confidence intervals.

Profession	2024	2025	2026	2027	2028	2029	2030
Medical Specialists Doctors	31355 (29787–32923)	32138 (30531–33745)	32942 (31295–34589)	33765 (32077–35453)	34610 (32880–36340)	35475 (33701–37249)	36362 (34544–38180)
Medical Doctors	24380 (23161–25599)	24989 (23740–26238)	25614 (24333–26895)	26254 (24941–27567)	26910 (25564–28256)	27583 (26204–28962)	28273 (26859–29687)
Non-physician Clinicians (HO)	30592 (29062–32122)	31356 (29788–32924)	32140 (30533–33747)	32944 (31297–34591)	33767 (32079–35455)	34612 (32881–36343)	35477 (33703–37251)
Nurses	103690 (98506–108874)	106282 (100968–111596)	108939 (103492–114386)	111662 (106079–117245)	114454 (108731–120177)	117315 (111449–123181)	120248 (114236–126260)
Midwives	30130 (28624–31636)	30883 (29339–32427)	31655 (30072–33238)	32446 (30824–34068)	33257 (31594–34920)	34089 (32385–35793)	34941 (33194–36688)
Medical Laboratory	38060 (36157–39963)	39011 (37060–40962)	39986 (37987–41985)	40986 (38937–43035)	42011 (39910–44112)	43061 (40908–45214)	44137 (41930–46344)
Anaesthesia Professionals	10308 (9793–10823)	10565 (10037–11093)	10829 (10288–11370)	11100 (10545–11655)	11378 (10809–11947)	11662 (11079–12245)	11954 (11356–12552)
Pharmacists	19431 (18459–20403)	19916 (18920–20912)	20414 (19393–21435)	20925 (19879–21971)	21448 (20376–22520)	21984 (20885–23083)	22534 (21407–23661)
Health Extension Workers	72816 (69175–76457)	74636 (70904–78368)	76502 (72677–80327)	78414 (74493–82335)	80375 (76356–84394)	82384 (78265–86503)	84444 (80222–88666)
Admin & Support Professionals (Degree)	16587 (15758–17416)	17001 (16151–17851)	17426 (16555–18297)	17862 (16969–18755)	18308 (17393–19223)	18766 (17828–19704)	19235 (18273–20197)
Admin & Support Staff (Diploma & Below)	99523 (94547–104499)	102011 (96910–107112)	104561 (99333–109789)	107175 (101816–112534)	109854 (104361–115347)	112601 (106971–118231)	115416 (109645–121187)
Total for the above professions	**476,872**	**488,788**	**501,008**	**513,533**	**526,372**	**539,532**	**553,021**
Net increase per year		**11916**	**12220**	**12525**	**12839**	**13160**	**13489**
% of net increase		**2%**	**2%**	**2%**	**2%**	**2%**	**2%**
Aggregate % change from the baseline		2%	5%	7%	9%	12%	14%

### 3.3 Health workforce needs and supply gap analysis

The gap analysis comparing projected health workforce supply with the needs based on the Essential Health Services Package (EHSP) revealed significant shortages across most job categories from 2024 to 2030 (see Table 4). In 2024, the largest deficits were noted among nurses (−36,773), medical specialists (−24,251), and medical laboratory professionals (−23,264). These gaps are expected to remain considerable in 2030, with deficits of −45,555 for nurses, −25,609 for medical specialists, and −24,233 for medical laboratory professionals. The Staff Availability Ratios (SARs) for these categories stayed below 70% throughout the period and fell below 50% for laboratory professionals. Medical specialists faced particularly severe constraints, with the SAR improving only slightly, from 23% to 30%.

**Table 4:** Projected needs-based requirements versus projected supply gap analysis for health workers.

Profession	2024				2025				2027				2030			
	Need (a)	Supply (b)	Gap (a-b)	SAR (b/a)	Need (a)	Supply (b)	Gap (a-b)	SAR (b/a)	Need (a)	Supply (b)	Gap (a-b)	SAR (b/a)	Need (a)	Supply (b)	Gap (a-b)	SAR (b/a)
**Medical Specialist Doctors**	**31355**	**7104**	**24,251**	**23%**	**32138**	**7745**	**24,393**	**24%**	**33,765**	**8986**	**24,779**	**27%**	**36362**	**10,753**	**25,609**	**30%**
**Medical Doctors**	**24380**	**14,222**	**10,158**	**58%**	**24989**	**16,216**	**8773**	**65%**	**26,254**	**20,080**	**6174**	**76%**	**28273**	**25,577**	**2696**	**90%**
**Non-physician Clinicians (HO)**	**30592**	**17,780**	**12,812**	**58%**	**31356**	**18,763**	**12,593**	**60%**	**32,944**	**20,667**	**12,277**	**63%**	**35477**	**23,377**	**12,100**	**66%**
**Nurses**	**103690**	**66,917**	**36,773**	**65%**	**106282**	**68,282**	**38,000**	**64%**	**111,662**	**70,929**	**40,733**	**64%**	**120248**	**74,693**	**45,555**	**62%**
**Midwives**	**30130**	**22,786**	**7344**	**76%**	**30883**	**24,225**	**6658**	**78%**	**32,446**	**27,013**	**5433**	**83%**	**34941**	**30,980**	**3961**	**89%**
**Anaesthesia Professionals**	**10308**	**4912**	**5396**	**48%**	**10565**	**5069**	**5496**	**48%**	**11,100**	**5372**	**5728**	**48%**	**11954**	**5801**	**6153**	**49%**
**Medical Laboratory**	**38060**	**14,796**	**23,264**	**39%**	**39011**	**15,692**	**23,319**	**40%**	**40,986**	**17,431**	**23,555**	**43%**	**44137**	**19,904**	**24,233**	**45%**
**Pharmacists**	**19431**	**18,281**	**1150**	**94%**	**19916**	**20091.06**	**−175**	**101%**	**20925**	**23,598**	**−2673**	**113%**	**22534**	**28,586**	**−6052**	**127%**
**Health Extension Workers**	**72816**	**64,206**	**8610**	**88%**	**74636**	**69,491**	**5145**	**93%**	**78,414**	**79,730**	**−1316**	**102%**	**84444**	**94,296**	**−9852**	**112%**
**Admin & Support Professionals (Degree)**	**16587**	**15,250**	**1337**	**92%**	**17001**	**16,057**	**944**	**94%**	**17,862**	**17,621**	**241**	**99%**	**19235**	**19,847**	**−612**	**103%**
**Admin & Support Staff (Diploma & Below)**	**99523**	**66,842**	**32,681**	**67%**	**102011**	**67,464**	**34,547**	**66%**	**107,175**	**68,668**	**38,507**	**64%**	**115416**	**70,383**	**45,033**	**61%**
**Total**	**476,872**	**313,097**	**163,775**	**66%**	**488,788**	**329,096**	**159,692**	**67%**	**513,533**	**360,095**	**153,438**	**70%**	**553021**	**404196**	**148825**	**73%**

Some cadres showed improving alignment between supply and need. Medical doctors reduced their deficit from −10,158 (58% of SAR) in 2024 to −2696 (90% of SAR) in 2030, while midwives narrowed down their deficit from −7344 to −3961 (76% of SAR to 89% of SAR). Health Extension Workers shifted from shortage to surplus, moving from −8610 (SAR 88%) to +9852 (SAR 112%). Pharmacists also transitioned to surplus by 2030 (SAR rising from 94% to 127%). Administrative and support professionals with degree-level training reached near balance by 2027 and a small surplus by 2030, whereas diploma-level administrative staff experienced widening gaps over time.

Overall, the national workforce deficit declined from −163,775 in 2024 to −148,825 in 2030, with total SAR improving from 66% to 73%. Despite this progress, major shortages persisted in critical clinical cadres, particularly nurses, medical specialists, and laboratory professionals (Table 4).

### 3.4 Financial feasibility analysis: comparative estimates of fiscal space and the costs of health workforce supply and needs

**Fig. 3:**
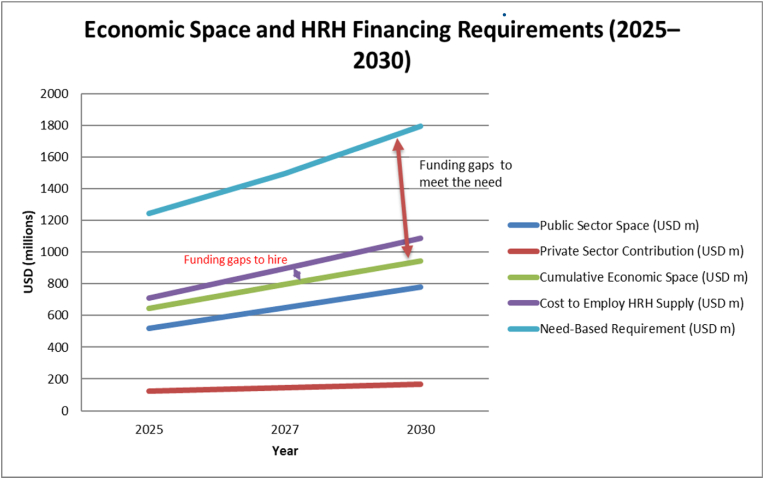
Economic feasibility analysis under different projection scenarios.

Fiscal space is currently inadequate to accommodate the projected workforce supply or fulfil the requirements based on the EHSP. By 2030, the cumulative economic space is estimated to reach USD 945 million, which falls short of the USD 1.08 billion needed to employ the expected workforce and the USD 1.8 billion required to fully meet the EHSP standards. Ongoing constraints on the wage bill suggest a persistent issue of graduate unemployment unless fiscal space expands. A summary of comparative cost estimates is provided in Table 5 and illustrated in Fig. 2, Fig. 3.

**Table 5:** Estimates of economic demand for health workers and different projection scenarios (USD in millions).

Variables	Estimate		
	2025	2027	2030
Public Sector Space (USD m) (A)	520	650	780
Private Sector Contribution (USD m) (B)	125	145	165
Cumulative Economic Space (USD m) (C)	645	795	945
Cost to Employ HRH Supply (USD m) (D)	709	898	1087
Need-Based Requirement (USD m) (E)	1245	1495	1795
Cost of training to fill population health needs-based gaps (USD in m)	594	647	742
Financial Gap (D–C) (USD m)	64	103	142
Gap to EHSP (E–C) (USD m)	600	700	850
Proportion of supply-side wage bill that could be absorbed by the estimated financial space (c/d)	91%	89%	87%
% Public Wage Needed to Absorb Unemployed	36%	38%	39%
Percent of financial space required to absorb “unemployed” health workers	13%	17%	21%
Overall EHSP as % of GDP	2.0%	1.9%	1.7%
HRH Supply as % of GDP	1.2%	1.3%	1.3%
Cumulative Space as % of GDP	1.1%	1.1%	1.0%
Absorb Gap as % of GDP	0.05%	0.05%	0.06%
EHSP Gap as % of GDP	1.0%	1.1%	1.2%

## 4 Discussion

This study provides a comprehensive analysis of Ethiopia's health workforce needs, supply prospects, and financial feasibility through 2030, revealing persistent mismatches between population health needs, labour market dynamics, and fiscal capacity. Despite moderate increases in the supply of most cadres, Ethiopia remains significantly below the WHO global threshold of 44.5 doctors, nurses and midwives per 10,000 populations [1] and the African Union's 2030 target of 36 per 10,000 populations [38]. This challenge mirrors trends across sub-Saharan Africa, where persistent health workforce shortages are projected to continue through 2030 despite ongoing training expansions [[39], [40], [41], [42]]. These findings reinforce the broader regional reality that increasing pre-service production without addressing structural constraints—such as financing, deployment imbalances, and retention—does not translate into improved workforce availability at the point of care.

The significant gaps between projected supply and needs in Ethiopia's health labour market reveal critical shortages, particularly among medical specialists, laboratory professionals, and nurses essential for clinical and diagnostic services. Similar trends are seen in Nigeria, Kenya, and Ghana, where physician shortages exceed 40% of national requirements and specialist production falls short of service delivery needs [43,44,45]. The shortage of medical specialists reflects a long-standing regional pattern in which generalist training expansion has outpaced specialist development. constraining referral-level and advanced care [46]. Ethiopia's predicted nursing gaps are consistent with global WHO projections indicating a deficit of 4.6 million nurses worldwide, disproportionately affecting low- and middle-income countries (LMICs) [36], and echo regional evidence showing that nurse shortages are a critical bottleneck to achieving UHC targets [47,48].

The projected deficits in medical laboratory professionals are consistent with evidence from Uganda, Tanzania, and Malawi, where shortages in diagnostic personnel undermine disease detection, treatment monitoring, and public health surveillance [49]. Given the growing importance of diagnostics within integrated disease surveillance and the One Health framework, these shortages pose a strategic risk to Ethiopia's health security agenda [50].

Conversely, the projected movement of health extension workers (HEWs) and pharmacists toward equilibrium or potential surplus mirrors experiences in Rwanda and Nepal, where sustained investments in community-level and mid-level cadres expanded primary care coverage [[45], [48], [51]]. Ethiopia's large-scale investment in community health workers and pharmacy training appears to be aligning production with demand. However, global evidence indicates that adequate numbers alone are insufficient; workforce performance depends on supportive supervision, enabling work environments, clear career pathways, and integration within multidisciplinary teams [51].

A critical and troubling finding of this study is the coexistence of persistent service delivery gaps with rising unemployment among newly trained health professionals. Despite unmet population health needs, fiscal constraints and wage-bill ceilings limit the public sector's capacity to absorb graduates, particularly among nurses, laboratory professionals, and selected mid-level cadres. This paradox—simultaneous workforce shortages and graduate unemployment—has been documented across several African countries, including Nigeria, Zambia, and Southern African states, where constrained public employment leads to underutilization of trained health workers [52]. Prolonged unemployment among new graduates' risks undermining morale, skills retention, and long-term workforce sustainability, as delayed entry into professional practice is associated with deskilling, migration, and declining returns on educational investment.

These dynamics are especially consequential for maternal and reproductive health outcomes. Ethiopia continues to face preventable mortality driven by shortages of competent, skilled birth attendants and emergency obstetric care providers. Evidence of competence gaps among graduating medical students, particularly in obstetric and emergency care, reflects limited clinical exposure, inadequate supervision, and overstretched training platforms [53]. Similar findings across Ethiopia and the region indicate that rapid expansion of health professional education without parallel investments in training quality, faculty, and clinical learning environments undermines both competence and employability [54,55]. Addressing HRH gaps for safe motherhood, therefore, requires not only increasing workforce numbers but also strengthening training quality and ensuring timely transition from education to employment.

Ethiopia's fiscal constraints remain the principal barrier to translating workforce supply into functional employment. By 2030, available fiscal space (USD 945 million) is insufficient to absorb the projected supply (USD 1.08 billion) and falls well short of the resources required to meet EHSP-based needs (USD 1.8 billion). Similar wage-bill constraints have been documented in Mozambique, Rwanda, Malawi, and Sierra Leone, where governments struggle to employ trained health workers despite service delivery deficits [56,57,58]. Ethiopia's public health expenditure—estimated at 4–6% of total government spending—remains far below the Abuja Declaration target of 15% [59]. These financing gaps directly contribute to graduate unemployment and signal a misalignment between education sector investments and health sector absorptive capacity.

Although Ethiopia's HRH strategic plan outlines evidence-based interventions—including incentive packages, rural retention strategies, competency-based training, career progression frameworks, and supportive supervision—implementation has been uneven [31,52,60]. International experience shows that HRH reforms yield results only when strategically sequenced, adequately financed, and closely monitored. Rwanda's performance-based financing and professional development initiatives improved retention and productivity [61], while Ghana's hardship allowances and structured career pathways enhanced rural distribution [[43], [44], [62], [63], [64]]. Ethiopia could similarly strengthen outcomes by linking workforce production more explicitly with employment planning, financing negotiations, and service delivery priorities.

### 4.1 Policy implications

The findings highlight the need for a coordinated, multi-sectoral response to Ethiopia's health labour market challenges. First, workforce planning should move beyond production targets toward explicit absorption planning, ensuring that pre-service education outputs are aligned with realistic public and private sector employment opportunities. Second, fiscal space for HRH must be expanded through efficiency gains, phased wage-bill negotiations, and strategic use of external financing to support graduate absorption, particularly in underserved areas. Third, targeted transition-to-practice mechanisms—such as paid internships, bonded rural deployment, and time-bound public service entry schemes—could mitigate graduate unemployment while addressing service gaps. Finally, sustained graduate unemployment risks weakening the education-to-employment pipeline by reducing the perceived return on investment in health professions, underscoring the importance of linking education policy, labour markets, and UHC reforms.

### 4.2 Conclusion

Ethiopia's health workforce supply is expanding but remains insufficient to meet population health needs and is increasingly constrained by limited fiscal space. The coexistence of persistent service delivery gaps and rising unemployment among newly trained health professionals reflects a fundamental misalignment between workforce production, financing, and employment capacity. Addressing these labour market mismatches through integrated planning, targeted graduate absorption strategies, and sustained financing reforms is essential for safeguarding workforce sustainability, protecting investments in health professional education, and achieving UHC and national and global health workforce targets by 2030.

## Patient and public involvement

Patients and/or the public were not involved in the design, or conduct, or reporting, or dissemination plans of this research.

## Patient consent for publication

Not applicable.

## Ethical considerations

The study utilized secondary data from public national and international databases, excluding individual-level data. While ethical clearance was not necessary, sources were properly acknowledged, and findings aim to inform policy dialogue in line with Ethiopia's national HRH strategy.

## Ethics statement

The descriptive and predictive health labour market analysis was undertaken as a *situation analysis* to inform the government's health workforce planning process. The analysis relied exclusively on secondary data and was not designed as primary research. It did not involve human participants, patients, or the collection of individual-level data; therefore, formal ethical approval was not required.

## Authors’ contributions

JM developed the study design, managed the data collection, performed the data analysis, interpreted the data, and wrote the manuscript. ZA, SS, FA, BB, and HD, contributed to interpretation of the data, and manuscript writing. FAD, TY, MY and JS contributed to the study design conception, data validation, interpretation of the data, wrote the manuscript and critical review of the manuscript before submission. All authors read and approved the final manuscript.

## Authors’ information

JMA: MA, MPH, Senior Human Resources for health management (HRHM) and leadership, management and governance (LMG) Advisor, University Medical Centre Groningen/University of Groningen. ZA: MPH, University Medical Centre Groningen/University of Groningen. SS: MD, MPH HRH Freelance Researcher; , TY: MD, MPH, PhD, HRH Consultant Mastercard Foundation, FAD, M.Sc. PhD Freelance Researcher Ethiopia; FA: HRH expert MOH, BB: HRH expert MOH, HD: Director HRMA MOH, MY: PhD Professor of Public Health Bahir Dar University Ethiopia JS: Professor, Department of Obstetrics and Gynecology, Leeuwarden Medical Centre and Department of Health Sciences, Global Health, University Medical Centre Groningen, University of Groningen, the Netherlands.

## Availability of data and materials

All data relevant to the study are included in the article. The datasets used and/or analyzed during the current study are available from the corresponding author on reasonable request.

## Consent for publication

Not applicable.

## Funding

Not available.

## Declaration of competing interest

We confirm that this manuscript has not been published elsewhere and is not under consideration by any other journal. We declare that there are **no conflicts of interest related to this manuscript**. All authors have approved the manuscript and agree with its submission to Public Health In Practice journal.
